# Voltage Dependent Potassium Channel Remodeling in Murine Intestinal Smooth Muscle Hypertrophy Induced by Partial Obstruction

**DOI:** 10.1371/journal.pone.0086109

**Published:** 2014-02-06

**Authors:** Dong-Hai Liu, Xu Huang, Xin Guo, Xiang-Min Meng, Yi-Song Wu, Hong-Li Lu, Chun-Mei Zhang, Young-chul Kim, Wen-Xie Xu

**Affiliations:** 1 Department of Physiology, Shanghai Jiaotong University School of Medicine, Shanghai, China; 2 Department of Physiology, Chungbuk National University College of Medicine, Cheongju, Chungbuk, Republic of Korea; Indiana University, United States of America

## Abstract

Partial obstruction of the small intestine causes obvious hypertrophy of smooth muscle cells and motility disorder in the bowel proximate to the obstruction. To identify electric remodeling of hypertrophic smooth muscles in partially obstructed murine small intestine, the patch-clamp and intracellular microelectrode recording methods were used to identify the possible electric remodeling and Western blot, immunofluorescence and immunoprecipitation were utilized to examine the channel protein expression and phosphorylation level changes in this research. After 14 days of obstruction, partial obstruction caused obvious smooth muscle hypertrophy in the proximally located intestine. The slow waves of intestinal smooth muscles in the dilated region were significantly suppressed, their amplitude and frequency were reduced, whilst the resting membrane potentials were depolarized compared with normal and sham animals. The current density of voltage dependent potassium channel (K_V_) was significantly decreased in the hypertrophic smooth muscle cells and the voltage sensitivity of K_V_ activation was altered. The sensitivity of K_V_ currents (IK_V_) to TEA, a nonselective potassium channel blocker, increased significantly, but the sensitivity of IKv to 4-AP, a K_V_ blocker, stays the same. The protein levels of K_V_4.3 and K_V_2.2 were up-regulated in the hypertrophic smooth muscle cell membrane. The serine and threonine phosphorylation levels of K_V_4.3 and K_V_2.2 were significantly increased in the hypertrophic smooth muscle cells. Thus this study represents the first identification of K_V_ channel remodeling in murine small intestinal smooth muscle hypertrophy induced by partial obstruction. The enhanced phosphorylations of K_V_4.3 and K_V_2.2 may be involved in this process.

## Introduction

Many congenital or acquired diseases often lead to hypertrophy of the tunica muscularis of the intestine, such as infantile hypertrophic pyloric stenosis, Hirschsprung's disease, achalasia and Chagas' disease [1]–[5]. Partial obstruction of the small intestine of mouse, rat, and guinea pig results in a notable hypertrophy of the intestinal wall due to thickening of the smooth muscle layer [6]–[9]. Hypertrophy of the gastrointestinal muscle wall, induced by partial obstruction, is a physiological response to the increased pressure in the lumen accompanied by motility disorder. The number and size of smooth muscle cells are increased within the hypertrophic intestinal wall [6]. The sensitivities of hypertrophic tunica muscularis to contractile and relaxing agents are also altered in rats [10]. In addition, the slow wave is deteriorated in hypertrophic segment and the resting membrane potential (RMP) of the intestinal smooth muscles is lower than control [6], [7].

It is well known that voltage-gated potassium (K_V_) channels are expressed in all gastrointestinal (GI) smooth muscle [11]. Based on their rates of activation and inactivation, two types of IK_V_ can be distinguished in mouse small intestinal smooth muscles: IK_V_ peak and IK_V_ sustained [11], [12]. IK_V_ peak reaches the maximum faster and inactivates slower than IK_V_ sustained [11], [12]. Although all K_V_ channels involve in regulating RMP, IK_V_ peak is activated at +10 mV∼+20 mV more negative than IK_V_ sustained and seems to be a major contributor to the resting membrane potential of smooth muscles [11], [13]. IKv peak is sensitive to 4-AP and resistant to TEA. On the contrary, IK_V_ sustained is a 4-AP resistant and TEA sensitive current [13]. According to the similar pharmacological and kinetic properties, the K_V_2 and K_V_4 families are thought to be K_V_ peak (K_Vpeak_) and K_V_ sustained (K_Vsustained_) in small intestine [14], [15]. These channels are the targets of many signaling pathways, including some kinases such as protein kinase A (PKA), protein kinase C (PKC), extracellular regulated protein kinases (ERK) and so on [16]–[20]. This means that, the function of the channels can be influenced by phosphorylation modification.

Apart from the reports indicating many inflammation-induced ion channels remodeling in the GI smooth muscle [21], [22], no information on the alterations of ion channels in smooth muscles (neither ionic currents nor the molecular basis) after hypertrophy of the intestine is available. The RMP of smooth muscles has been reported to decrease in hypertrophic small intestine, and the IK_V_ channels play an important role in regulating the RMP. Base on this information, we hypothesize that there is IK_V_ remodeling after hypertrophy in small intestinal smooth muscles, and this change involves depolarization of RMP. In the present study, we used an intestinal partial obstruction mouse model to explore the electrical remodeling and possible mechanisms involved in the motility disorder associated with obstruction-induced smooth muscle hypertrophy.

## Materials and Methods

### Ethics statement

Animals were obtained from the Experimental Animal Center of Shanghai Jiaotong University School of Medicine. This study was carried out in strict accordance with the recommendations in the Guide for the Care and Use of Laboratory Animals of the Science and Technology Commission of P.R.C. (STCC Publication No. 2, revised 1988). The protocol was approved by the Committee on the Ethics of Animal Experiments of Shanghai Jiaotong University School of Medicine (Permit Number: Hu 686-2009). All surgery was performed under ether, and all efforts were made to minimize suffering.

### Animal model and tissue preparation

Male imprinting control region (ICR) mice aged between 5–6 weeks and with a body weight of 30±4 g were used. Small bowel obstruction was induced by tightening a ring around the ileum as describe before [6]. Briefly, mice were anaesthetized with chloral hydrate (300 mg/kg, I. p.), and all efforts were made to minimize suffering. Partial intestinal obstruction was induced by surgically placing a ring (5 mm in length, 4 mm external diameter, 3 mm internal diameter) of silicon tube around the ileum 30–50 mm oral to the ileocecal sphincter to cause distension of the intestine proximal to the site of obstruction. Experiments were performed with the distended segments of ileum 14 days after surgery [6]. Sham operations were performed using the same surgical exposure procedures except the ileal obstruction. Mice were anaesthetized with ether and euthanized by cervical dislocation. A 60-mm segment of small intestine oral to the obstruction was removed and pinned out in the base of a Sylgard silicone elastomer dish containing Krebs of the following composition: NaCl 118.5 mM; KCl 4.5 mM; MgCl_2_ 1.2 mM; NaHCO_3_ 23.8 mM; KH_2_PO_4_ 1.2 mM; glucose 11.0 mM; CaCl_2_ 2.4 mM. The intestinal segment was opened by cutting lengthwise, washed with Krebs, and the mucosa and submucosa were removed by sharp dissection. The remaining tunica muscularis was used for all experiments.

For Western blot and immunoprecipitation experiments, the tunica muscularis was quickly frozen in liquid nitrogen. Samples were homogenized in ice-cold RIPA buffer [25 mM Tris-HCl pH 7.6, 150 mM NaCl, 1% NP-40, 1% sodium deoxycholate, 0.1% SDS and phosphatase and protease inhibitors (20 mM β-glycerophosphate, 20 mM sodium pyrophosphate, 50 mM NaF, 1 mM each of EDTA, EGTA, sodium orthovanadate, p-nitrophenyl phosphate, Phenylmethanesulfonyl fluoride (PMSF) and benzamidine, and 5 µg/ml each of aprotinin, leupeptin and pepstatin A)]. The homogenates were centrifuged at 800 g for 10 min at 4°C for Western blot, or at 12000 g for 15 min at 4°C for immunoprecipitation. The supernatants were collected. Protein concentrations were determined by Bradford method (Pierce, Rockford, IL, USA). Samples were stored at −80°C until use.

### Immunoprecipitation and Western blot analysis

For immunoprecipitation, the protein samples (each containing 400 µg of protein) were diluted four folds with RIPA buffer. Samples were preincubated for 1 h with 20 µL protein G-sepharose (Beyotime, Jiangsu, China) and then centrifuged to remove any protein adhered non-specifically to the protein G-sepharose. The supernatant was incubated with 5 µg proper antibodies for 4 h at 4°C. After the addition of protein G-sepharose, the mixture was incubated at 4°C for an additional 2 h. Samples were triple washed with RIPA buffer and eluted by sodium dodecyl sulfate-polyacrylamide gel electrophoresis (SDS–PAGE) loading buffer then boiling at 98°C for 5 min. Western blot analysis was carried out on 10% SDS–PAGE. Briefly, proteins were electrotransferred onto Immobilon-p PVDF membrane (millipore, Billerica, MA, USA, pore size 0.45 µm). After blocking for 1 h in Tris-HCl-buffered saline with 0.1% tween-20 (TBST) and 5% non-fat milk, the membranes were incubated over night with primary antibody in TBST containing 5% non-fat milk. Detection was carried out by the use of proper horseradish peroxidase conjugated second antibodies and developed with beyoECL plus assay kit (Beyotime chemical Co., Jiangsu, China).

### Intracellular microelectrode recording

Strips of tunica muscularis (8 mm×4 mm) were cut parallel to the longitudinal axis of the intestine, oral to the site of occlusion. The muscle strips were pinned onto the base of a Sylgard-coated chamber, circular muscle side up, and continuously perfused with warmed (37°C, 1.2 ml/min) and oxygenated Krebs solution. Stripes were equilibrated for approximately 2 h before recording. Cells were impaled with KCl-filled glass microelectrodes with resistances of 50–90 MΩ. Electrical responses were recorded and amplified through a high input impedance amplifier (SYS-773 Duo 773 Electrometer, WPI, USA). Experiments were performed in the presence of nifedipine (1 µM; Sigma, St Louis, MO, USA) in the perfusion solution to reduce contraction and facilitate cell impalement. Slow waves in mouse intestine have been previously shown to be unaffected by nifedipine [23].

### Cell preparation and Voltage patch-clamp

Smooth muscle cells were prepared from the small intestinal region as described above. After the mucosal, submucosal layers were carefully removed, the tunica muscularis was cut into small segments (1×4 mm). These segments were kept in modified Kraft–Bruhe (KB) solution (EGTA 0.5 mM, Hepes 10 mM, MgCl_2_ 3 mM, KCl 50 mM, glucose 10 mM, KH_2_PO_4_ 20 mM, taurine 20 mM, and glutamic acid 50 mM, adjusted to pH 7.4 with KOH) for 15 min at 4°C. They were then incubated at 37°C in 1 ml of digestion medium (Ca^2+^-free solution) containing 1.5–2 mg collagenase II or collagenase I (Worthington Biochemicals, Lakewood, NJ, USA), 400–600 µg papain (Sigma-Aldrich, St. Louis, MO, USA), 1.5 mg dithiothreitol, 1.5 mg trypsin inhibitor (Amresco Inc., Solon, OH, USA), and 3 mg bovine serum albumin (Sigma-Aldrich, St. Louis, MO, USA) for 10–15 min. The Ca^2+^-free solution contained NaCl 135 mM, KCl 5 mM, MgCl_2_ 1.2 mM, glucose 10 mM, and Hepes 10 mM, adjusted to pH 7.4 with Tris. After digestion, the supernatant was discarded and the muscle segments were washed with the modified KB solution. Tissue pieces were gently triturated using a wide-bore fire-polished glass pipette (2 mm bore diameter) to create a cell suspension. Dispersed cells were kept in modified KB solution at 4°C for later use. Drops of the cell suspension were transferred to a perfusion chamber on the stage of an inverted microscope, and allowed to adhere to the bottom of the chamber for 20–25 min prior to recording. The cells were then continuously perfused with physiologic saline solution (PSS, NaCl 135 mM, KCl 5 mM, CaCl_2_ 2 mM, MgCl_2_ 1.2 mM, glucose 10 mM, and Hepes 10 mM, adjusted to pH 7.4 with Tris). A single 4-channel perfusion system (BPS-4, ALA Inc., Westbury, NY, USA) was used to change the perfusate. The whole cell patch-clamp technique was used to record the voltage-dependent K^+^ currents (IK_V_) with an EPC-10 amplifier (HEKA Elektronik, Lambrecht, Germany). Data were filtered at 10 kHz, recorded using patchmaster software (HEKA Elektronik, Germany) and digitized at 20 kHz. In cases of long records the data were sampled at 10 kHz. Capacitance was compensated and residual capacitance current was digitally removed. Series resistance was not compensated for. Series resistance was between 2 and 4 MΩ. Cell membrane capacitance was calculated from the time constant of a capacitance current elicited by a 5 mV depolarization from −60 mV. Pipette resistances were 2–5 MΩ. The pipette solution comprised KCl 20 mM, potassium-aspartic acid 110 mM, di-tris-creatine phosphate 2.5 mM, MgATP 5 mM, Hepes 5 mM, MgCl_2_ 1.0 mM, and EGTA 10 mM, adjusted to pH 7.3 with Tris. All current amplitudes were normalized to the cell membrane capacitance (Cm) and expressed as densities (pA/pF). In some experiments 4-AP or TEA were added to the bath. All experiments were performed at room temperature (20–25°C). The peak value of IKv was measured as IK_Vpeak_, the mean value of last 100 ms of depolarization was measured as IK_Vsustained_ to minimize the IK_Vpeak_ influence. The voltage dependence of the inactivation was fit by the Boltzmann function: *I*/*I*max = 1/{1+exp [(V−V_0.5_)/k]}. Where *I* is the amplitude of IKv, V_0.5_ is the half-activation voltage and k is the slop factor at this voltage. Voltage-dependent activation of IKv was respectively converted into conductivity using the Goldman-Hodgkin-Katz current equation [14]. Goldman-Hodgkin-Katz current equation: 
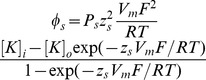
. 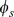 is the IKv amplitude, *P_S_* is the permeability of the membrane, z_s_ is the valence of ion potassium, V_m_ is the transmembrane potential, F is the Faraday constant, equal to 96,485 C·mol−1, R is the gas constant, equal to 8.314 J·K−1·mol−1, T is the absolute temperature, measured in Kelvin, [K]_i_ is the intracellular concentration of K^+^ (130 mM, concentration of pipette solution), [K]_o_ is the extracellular concentration of K^+^ (5 mM, concentration of physiologic saline solution). The voltage dependence of the activation was fit by the Boltzmann function: *P_S_*/*P_S_*_max_ = 1/{1+exp [−(V−V_0.5_)/k]}. V_0.5_ is the half-inactivation voltage and k is the slop factor at this voltage.

### Immunofluorescence

Small intestine tissues were collected after animals were killed by cervical dislocation. The samples were fixed with 4% paraformaldehyde (PFA) overnight at 4°C and sectioned at 5 µm thickness. After that, the sections were blocked with 10% goat serum in phosphate-buffered saline (PBS) for 1 h at room temperature. To check for Kv4.3 and Kv2.2 immunoreactivity, the sections were incubated with mouse anti–Kv4.3 antibody (1∶1000) and mouse anti–Kv2.2 antibody (1∶1000) overnight at 4°C, respectively. The sections were incubated with goat anti-mouse antibody (1∶1000) conjugated with DyLight488 for 1 h at room temperature followed by nucleic acid dye DAPI and membrane dye Fm 4-64 membrane stain (Invitrogen, Eugene, Oregon, USA). Pictures were acquired by a confocal laser-scanning microscope (Olympus FV-1000, Japan).

### Antibodies and Drugs

The following primary antibodies were used: anti-Kv4.3 (ab99045, for Western blot) and anti-Kv2.2 (ab10651) were acquired from Abcam (Hong Kong, China). Anti-Kv2.2 (sc-292489, for immunoprecipitation) and anti-p-Ser (sc-80514) antibodies were acquired from Santa Cruz Biotechnology (Santa Cruz, CA, USA). Anti-p-Ther antibody (#9386) was obtained from cell signaling technology (City and country of the company). The secondary antibody DyLight488 conjugated goat anti-mouse IgG (16117031711) was bought from ImmunoReagents, Inc. (Raleigh, NC, USA). The secondary antibody horseradish peroxidase (HRP) conjugated goat anti-mouse IgG (sc-2005) was bought from Santa Cruz Biotechnology (Santa Cruz, CA, USA). Tetraethylammonium chloride (TEA), 4-Aminopyridine (4-AP) and other chemicals were all acquired from Sigma unless indicated otherwise.

### Statistical Analysis

Data of Western blot were reported as the mean±SD, other data were reported as mean±S.E.M.; n refers to the number of animals from which tissues were obtained. Statistical analysis of the results was carried out by one-way analysis of variance (ANOVA) followed by the least significant difference test or Newman–Keuls test. Differences of p<0.05 were considered significant. Semiquantitative analysis of the bands was performed with the Image J analysis software (Version 1.30v; Wayne Rasband, NIH, USA). Data analysis was performed with SigmaStat Software (Version 3.5, Jandel Scientific Software, California, USA). Curve fitting were performed with SigmaPlot Software (Version 10.0, Jandel Scientific Software, California, USA) and GraphPad Prism 6 (GraphPad Software, La Jolla, USA)

## Results

### 1. Alteration of intestinal smooth muscle cells membrane capacitance and RMP

**Figure 1:**
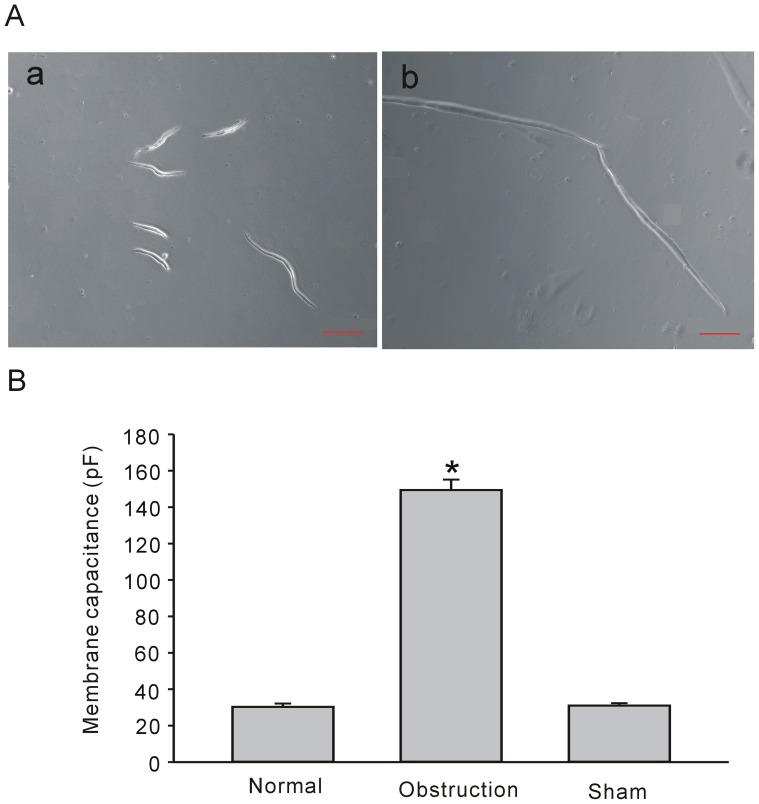
The hypertrophy of smooth muscle cells induced by partial intestinal obstruction. A shows normal (a) and hypertrophic smooth muscle cells (b), the cell size was significantly enlarged in obstruction group. B shows the mean values of cell capacitances among normal, sham and obstruction groups. Data showed the means ± SE *P<0.01 versus sham and normal groups, *P<0.05 versus normal and sham group, Bar = 100 µm.

In order to determine the histological and electrical remodeling and further confirm the hypertrophic state of freshly dissociated intestinal smooth muscle cells in partial intestinal obstruction model, we compared the cell capacitance among normal, sham, and obstruction groups. The cell size was significantly enlarged in obstruction group (Fig. 1A). The membrane capacitance of obstruction group (149.37±5.74 pF; n = 40; P<0.05) was significantly increased compared with sham (30.99±1.33 pF; n = 36) and normal groups (30.32±1.84 pF; n = 35) (Fig. 1B).

**Figure 2:**
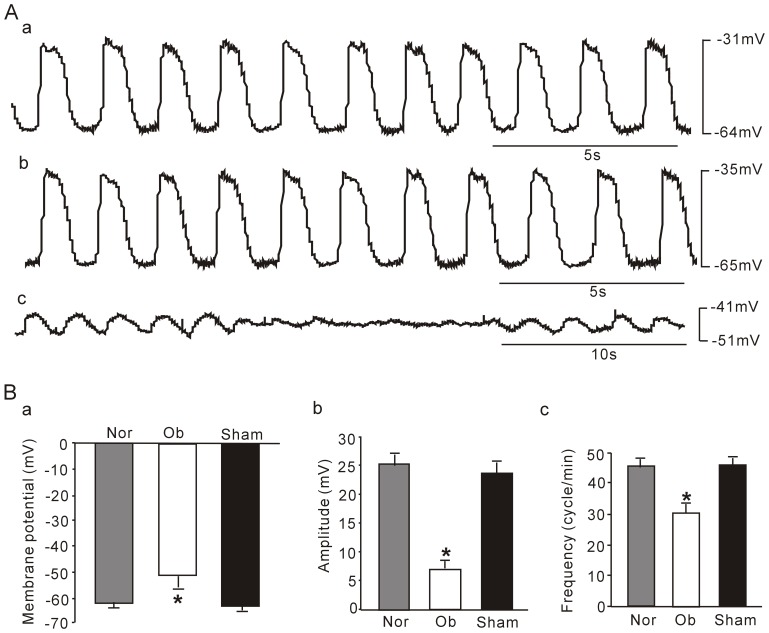
Changes of slow wave and resting membrane potential (RMP) in hypertrophic smooth muscles. Electrical slow waves were recorded from small intestinal muscle stripes of normal group (Aa), sham group (Ab), and obstruction group (Ac). The RMP (Ba), amplitudes (Bb) and frequencies (Bc) of slow wave were significantly changed in normal, sham and obstruction groups. Data showed the means ± SE *P<0.01 versus sham and normal groups.

The intracellular microelectrode recording method was utilized to record the slow wave and RMP in muscle strips in normal, sham and obstructed intestinal smooth muscle strips to further confirm whether histological remodeling is accompanied by electric remodeling. Our observation was similar to previous reports [6], [7]. The RMP as well as the amplitude and frequency of slow wave were all decreased after obstruction (Fig. 2A). The average RMPs were −62.4±5.04 mV, −63±5.24 mV and −51.33±11.88 mV*, respectively, in normal, sham and obstruction groups (Fig. 2B, a, n_normal_ = 6, n_sham_ = 7 and n_obstruction_ = 7, respectively, *P*<0.05). The average amplitude and frequency were 25.42±1.4 mV, 23.82±1.6 mV, 7.25±1.3 mV* and 45.6±1.25 cycle/min, 46.1±1.18 cycle/min and 30.44±1.3 cycle/min*, respectively, in normal, sham and obstruction groups (Fig. 2B, b, c, n_normal_ = 6, n_sham_ = 7 and n_obstruction_ = 7, respectively, *P*<0.05). These findings suggest that histological remodeling in intestinal obstruction is accompanied by electric remodeling, which may lead to motility disorders.

### 2. IK_V_ densities and properties in hypertrophic intestinal smooth muscle cells

**Figure 3:**
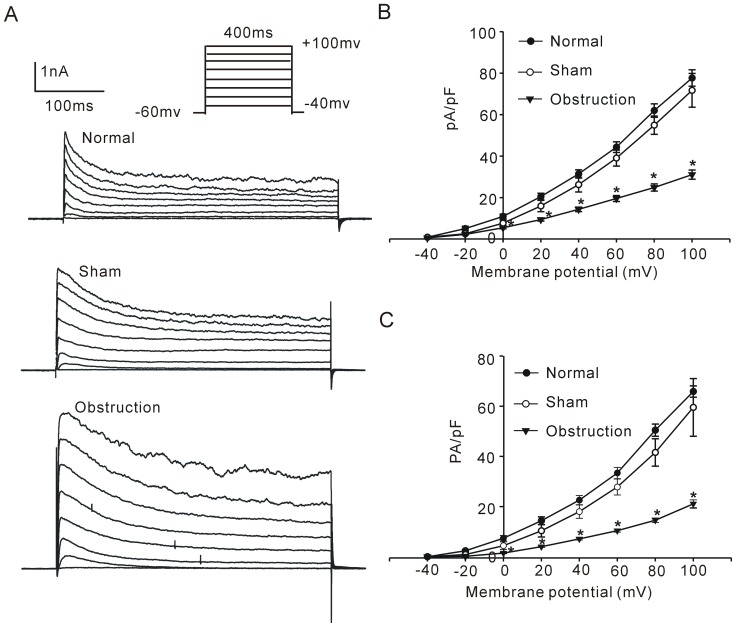
Comparison of IK_V_ in normal sham and obstruction groups. A shows representative current traces elicited from a holding potential of −60 mV using voltage steps of 400 ms from −40 mV to +100 mV in 20 mV increments. Interval between each pulse is 10 s. Averaged current density-voltage relation of IK_Vpeak_ (B) and IK_Vsustained_ (C) plotted for the smooth muscle cells from normal, sham, and obstruction groups. Data showed the means ± SE *P<0.01 versus sham and normal groups.

IK_V_ can be separated into two components by its sensitivities to TEA and 4-AP, two traditional potassium channel blockers. We studied changes of the two IK_V_ components in the hypertrophic intestinal smooth muscle cells. Fig. 3A showed that incremental 20 mV depolarizing steps with 400 ms duration from a constant holding potential of −60 mV to test voltages as positive as +100 mV elicited families of outward currents in intestinal smooth muscle cells of normal, sham and obstruction groups. The average densities of peak potassium currents from beginning to100 ms (IK_Vpeak_) and the mean currents from 300 ms to the end (IK_Vsustained_) elicited at each test potential were calculated to compare current-voltage (I–V) relationships in normal, sham and obstruction groups. The densities of IK_Vpeak_ and IK_Vsustained_ in hypertrophied smooth muscle cells was higher than that in normal and sham groups at every commend membrane potential from 0 mV to +100 mV (Fig. 3B). For example, the current densities of IK_Vpeak_ elicited by step depolarizing pulses to +40 mV and +60 mV were 31.25±0.978 pA/pF, 44.17±2.57 pA/pF in normal group, 26.06±3.51 pA/pF, 38.89±3.84 pA/pF in sham group and 14.18±1.07 pA/pF, 19.41±1.45 pA/pF in obstruction group, respectively (Fig. 3B, n_normal_ = 13, n_sham_ = 11 and n_obstruction_ = 12, respectively, *P*<0.05 *vs* normal or sham group). The current densities of IK_Vsustained_ elicited by step depolarizing pulses to +40 mV and +60 mV were 22.57±1.83 pA/pF, 33.36±2.26 pA/pF in normal group, 17.97±2.65 pA/pF, 27.79±3.17 pA/pF in sham group and 7.10±0.43 pA/pF, 10.38±0.58 pA/pF in obstruction group, respectively (Fig. 3C, n = 13, 11 and 12, respectively, *P*<0.05 *vs* normal or sham group). The results suggest that the IK_V_ function is down-regulated in the hypertrophic intestinal smooth muscle cells induced by partial obstruction.

**Figure 4:**
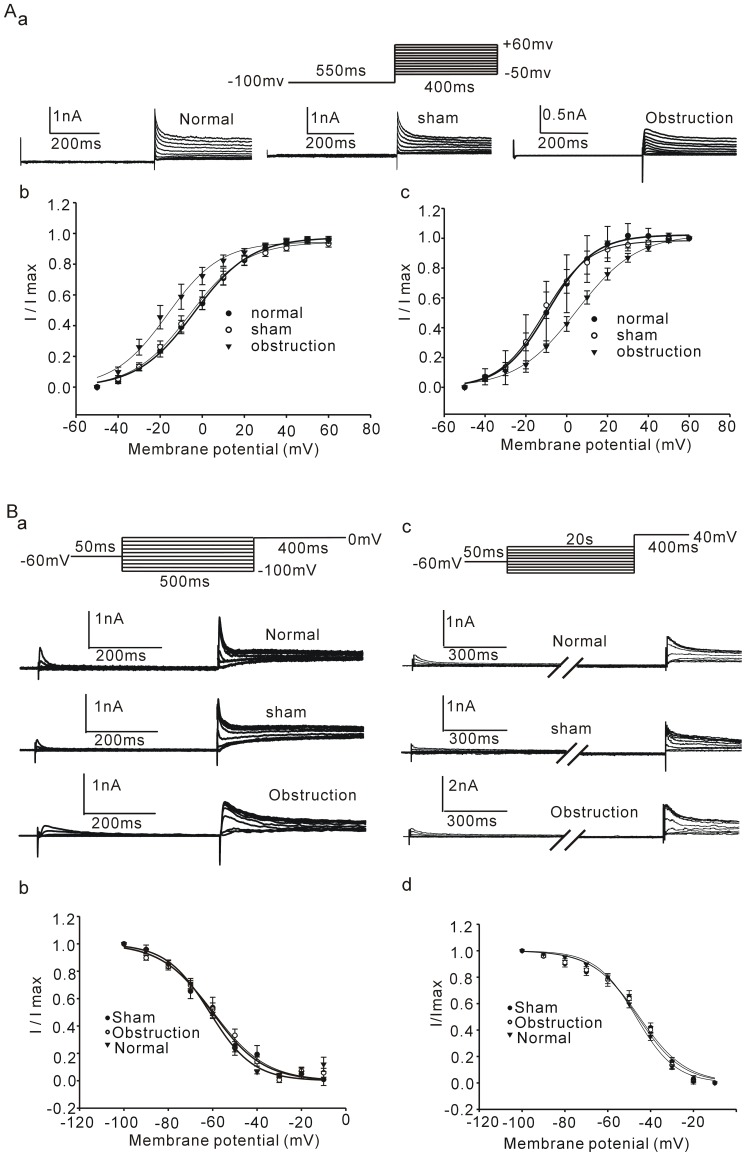
Comparisons of voltage-dependent activation and inactivation of IK_V_ in normal, sham and obstruction groups. Aa shows representative raw traces were elicited by a series of voltage champs from a holding potential of −60 mv to selected test potentials ranging from −50 to +60 mV for 400 ms. Interval between each pulse is 10 s. Voltage-dependent activation of IK_Vpeak_ (Ab) and IK_Vsustained_ (Ac) were respectively converted into conductivity using the Goldman-Hodgkin-Katz current equation. The conductivies were the normalized and plotted as a function of test potential and fitted with a Boltzmann function. Ba shows representative current traces of IK_V_ were elicited by a series of the conditioning potential ranging from −100 to 0 mV for 500 ms following a 0 mV test potential for 400 ms. Bc shows current traces of IK_V_ were elicited by a series of the conditioning potential ranging from −100 to 0 mV for 20 s following 40 mV test potential to adequately activate the IK_Vsustained_. Interval between each pulse is 10 s (Ba c). Voltage-dependent inactivation curves of IK_Vpeak_ (Bb) and IK_Vsustained_ (Bd) were plotted as a function of the conditioning potential and fitted with a Boltzmann function. Data showed the means ± SE(Ab c, Bb c).

In succession the steady-state activation and steady-state inactivation curves were obtained in the normal, sham and obstructed intestinal smooth muscle cells to determine whether the down regulation of current densities is associated with alteration of channel voltage sensitivity (Fig. 4). The shifts of activation curves of IK_Vpeak_ and IK_Vsustained_ showed different tendency in hypertrophic smooth muscle cells. In comparison to sham and normal groups, the half-activation voltage (V_0.5__act_) of IK_Vpeak_ in hypertrophic smooth muscle cells shifted to more negative potential (V_0.5__act_, normal = −2.78±2.64 mV, sham = −4.3±3.63 mV and obstruction = −15.74±3.8 mV, n = 12, 11, 13, respectively, *P*<0.05, Fig. 4A, a, b). On the contrary, the half-activation voltage of IK_Vsustained_ shifted to more positive potential (V_0.5_ act, normal = −8.65±2.78 mV, sham = −8.99±3.8 mV and obstruction = 5.14±2.61 mV, n = 12, 11, 13, respectively, *P*<0.05, Fig. 4A, c). However, the half-inavtivation voltage (V_0.5inact_) of IK_Vpeak_ slightly shifted to positive in hypertrophic smooth muscle cells but there were no significant difference among normal, sham and obstruction groups (Fig. 4B, a, b, V_0.5_ inact, normal = −61.2±2.27 mV, sham = −60.1±2.88 mV and obstruction = −53.55±3.79 mV, n = 12, 11, 12, respectively). Besides of this, no significant alteration was observed in the slop factors and IK_Vsustained_ inactive curves among obstruction, normal and sham groups (Fig. 4 A, a, b, c, B, a, b, c, d, V_0.5inact_ (normal) = −46.38±3.94 mV, V_0.5inact_ (sham) = −47.52±3.58 mV and V_0.5inact_ (obstruction) = −47.24±4.08 mV, n = 10, 9, 11, respectively).

### 3. Effect of TEA and 4-AP on IK_V_

**Figure 5:**
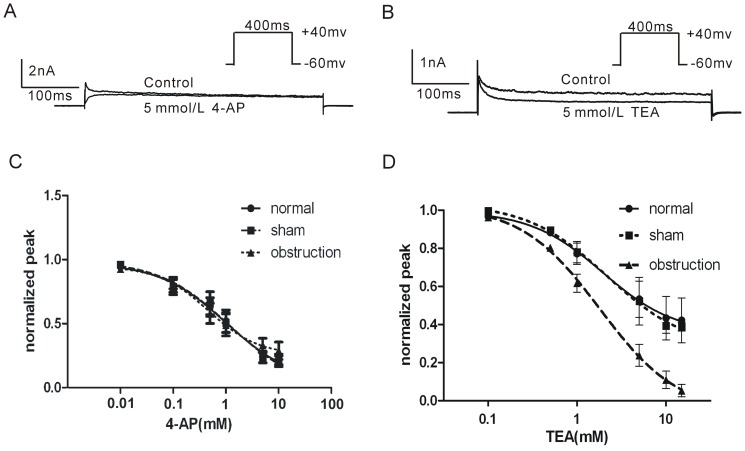
Comparison of the sensitivities of IK_V_ to 4-AP and TEA in normal, sham and obstruction groups. Membrane currents elicited by 400−60 mV to +40 mV in control or in 4-AP 5 mM (A) or TEA 5 mM (B). Interval between each pulse is 10 s. Average dose-response curves of 4-AP (C) and TEA (D) where each point was the averaged I_test_/I_control_ and error bars were means ± SE. IC_50_ values were obtained through the software GraphPad Prism 5.

4-AP and TEA are two kinds of voltage-dependent potassium channel blockers that have been widely used in studying K_V_ channel function. In order to examine the sensitivities of IK_Vpeak_ and IK_Vsustained_ to 4-AP and TEA, we compared the dose-response curves of 4-AP and TEA in intestinal smooth muscle cells of normal, sham and obstruction groups, respectively. The results demonstrated that IK_Vpeak_ was more sensitive to 4-AP (5 mM) and IK_Vsustained_ was more sensitive to TEA (5 mM) when the membrane potential was depolarized from −60 mV of holding potential to +40 mV (Fig. 5A and B). There was no significant difference in IK_V_ sensitivity to 4-AP among obstruction, normal and sham groups (Fig. 5C; *P*>0.05). However, the IC_50_ of TEA on IKV in hypertrophic smooth muscle cells was slightly decreased than that in normal and sham groups (Fig. 5D, *P>0.05*). Interestingly, when the membrane potential was depolarized from −60 mV of holding potential to +40 mV, TEA (15 mM) almost completely blocked the IK_Vsustained_ in hypertrophic smooth muscle cells (95.3±0.04%), but TEA only blocked 55.4±0.05% and 52.7±0.05% of the IK_Vsustained_ in normal and sham groups (Fig. 5D). These results suggest that the sensitivity of IK_Vsustained_ to TEA is significantly increased in hypertrophic smooth muscle cells.

### 4. Alteration of K_V_ channel subunits

**Figure 6:**
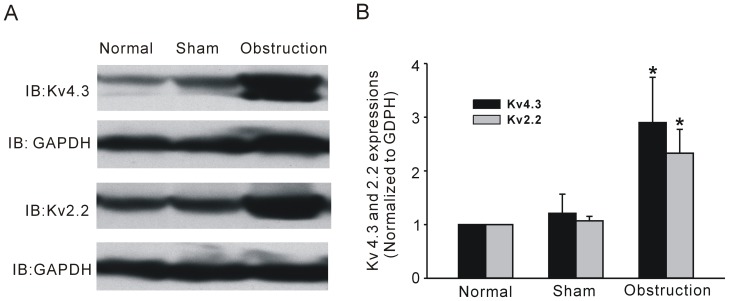
Kv4.3 and Kv2.2 expressions of intestinal smooth muscle tissues in normal, sham, and obstruction groups. A shows the westernblot bands performed with anti-Kv4.3 or anti- Kv2.2 antibodies to detect the Kv4.3 and Kv2.2 expression levels. GAPDH was used as internal control to normalize for difference in loading. Corresponding bands were scanned and the Kv4.3 or Kv2.2 band optical density was normalized by the GAPDH protein density. Data showed the means ± SD, n = 6, *P<0.05 versus sham groups (B).

**Figure 7:**
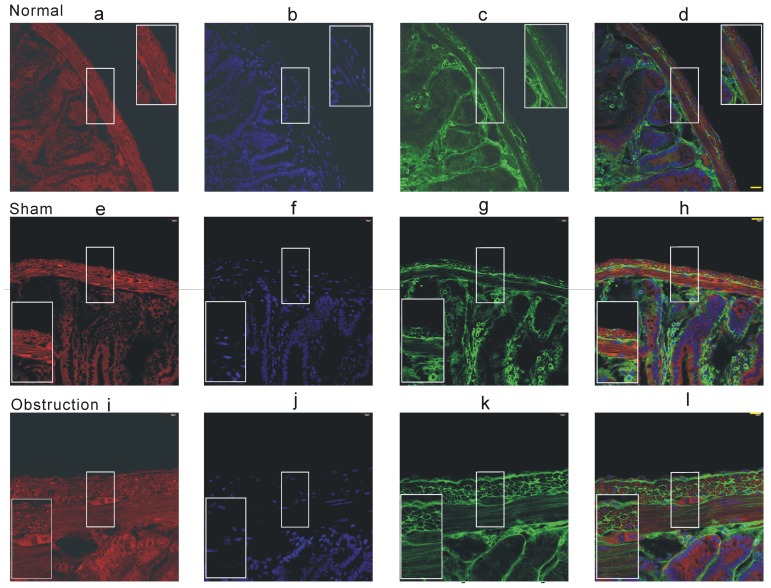
Immunofluorescence staining of K_V_4.3 in the mice small intestine frozen section. The cell membrane was stained by Fm 4-64 membrane stain (red fluorescence) and cell nuclear was stained by DAPI (blue fluorescence). The green fluorescence was KV4.3 immunofluorescence. Scale bar = 20 µm, the experiments repeated six times.

**Figure 8:**
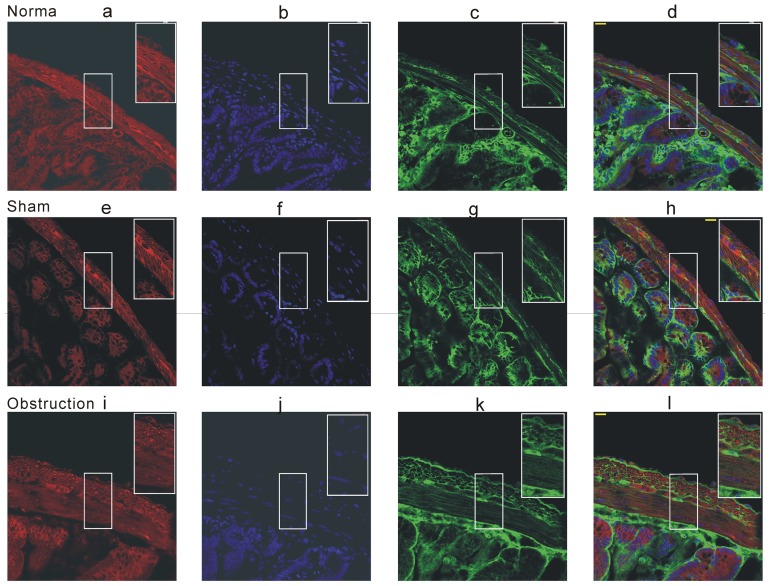
Immunofluorescence staining of K_V_2.2 in the mice small intestine frozen section. The cell membrane was stained by Fm 4-64 membrane stain (red fluorescence) and cell nuclear was stained by DAPI (blue fluorescence). The green fluorescence was K_V_2.2 immunofluorescence. Scale bar = 20 µm, the experiments repeated six times.

In further experiments, we examined whether K_V_ channel pore forming α subunit alteration could be correlated with partial obstruction-induced electric remodeling. Western blot analysis was performed to observe alteration of channel subunit protein expression. Two immunoreactive bands were detected at ≈71 kD and at ≈102 kD respectively, the former is corresponding to the long isoform of Kv4.3 channel protein and the latter is the Kv2.2 band (Fig. 6A). The expression levels of both Kv4.3 and Kv2.2 were up-regulated in hypertrophic intestine smooth muscle tissues compared with sham and normal tissues (Fig. 6B, n = 6, *P*<0.05). The result was quite opposite to the change in IK_V_ densities. To further confirm the Western blot result, we next compared the protein expression in situ with the immunofluorescence method. Similar results of increased Kv4.3 and Kv2.2 immunoreactivities were observed in frozen sections (Fig. 7 and Fig. 8). In the normal and sham groups, weak Kv4.3 and Kv2.2 immunoreactivities were detected in the smooth muscle regions of small intestine, but the change in mucosa layer was not found. Two weeks after ileum obstruction, Kv4.3 and Kv2.2 fluorescence intensity was significantly increased compared with the normal and sham groups (Fig. 7 and Fig. 8). In addition, with the cell membrane staining, we found that the most channel immunostaining was merged with plasmalemma of longitudinal muscle cells (Fig. 7 and Fig. 8). This told us that channel proteins were still in the plasmalemma other than blocked in the ion channel trafficking pathway.

**Figure 9:**
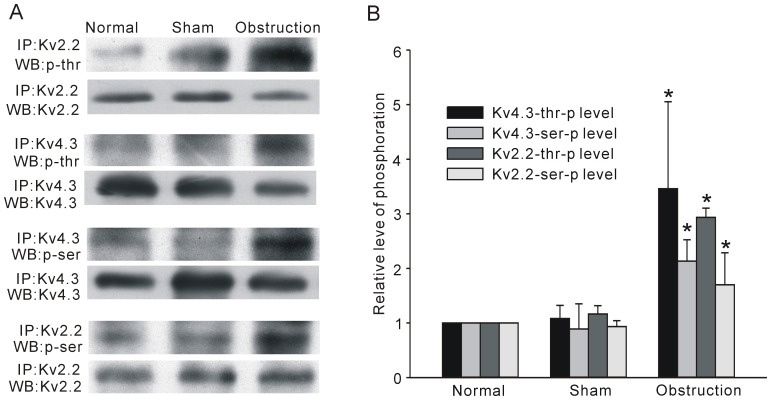
Phosphorylation levels of K_V_4.3 and K_V_2.2 in normal, sham, and obstruction groups. Phosphorylation of Kv4.3 and Kv2.2 were examined by immunoprecipitation (IP) with ant-Kv4.3 antibody followed by IB with antibody against p-threonine, p-serine and Kv4.3 or IP with anti- Kv2.2 antibody followed by IB with antibody against p-threonine, p-serine and Kv2.2 (A). Corresponding bands were scanned and the phosphorylation band optical density was normalized by the total protein density. Data were the means ± SD and were expressed as folds versus normal. *P<0.05 versus normal and sham, n = 5 (B).

Protein phosphorylation in particular plays a significant role in regulating protein function. Thus, we examined changes in channel protein phosphorylation level for Kv4.3 and Kv2.2. Because no commercial antibodies against Kv4.3 specific phosphorylation sites are available and there are no reports of phosphorylation about the Kv2.2, we pulled down the channel proteins from the intestinal smooth muscles tissue with immunoprecipitation method and used the anti-p-threonine or anti-p-serine antibodies to detect the whole phosphorylation levels of immunoprecipitated proteins. As shown in Fig. 9, in the protein pulled down by anti-Kv2.2 antibody, the ratios of phosphorylated threonine protein and phosphorylated serine protein to total protein were up-regulated significantly in obstruction group compared with other two groups (Fig. 9A and B, n = 5, *P*<0.05). The ratios of phosphorylated protein to total protein of Kv4.3 were also increased in hypertrophic smooth muscle tissue (Fig. 9A and B). These results suggested that there were great alterations of K_V_ channel phosphorylation in hypertrophic segment of small intestine.

## Discussion

In the present study, we investigated the voltage dependent potassium channel remodeling in hypertrophic smooth muscle cells induced by partial intestinal obstruction in mouse. Our experiments demonstrated that partial obstruction-induced smooth muscle hypertrophy in the proximally located intestine after 14 days of obstruction was accompanied by electric remodeling. The amplitude and frequency of slow waves of intestinal smooth muscles were significantly reduced, whilst the resting membrane potentials were depolarized compared with normal and sham animals. The current density of IK_V_ was profoundly reduced. The steady-state activation curve of IK_Vpeak_ was shifted to the negative potential and the IK_Vsustained_ was shifted to the positive potential in hypertrophic smooth muscle. However, no significant difference was observed in the inactive curves of IK_V_. The sensitivity of IK_Vsustained_ to TEA was also increased in hypertrophic smooth muscle cells. Interestingly, the expressions of the main isoforms, pore-forming Kv4.3 and Kv4.2 subunits were up-regulated in the hypertrophic smooth muscle cells membrane. In addition, both threonine and serine phosphorylation levels in Kv4.3 and Kv4.2 channel proteins were obviously up-regulated. The data implies that phosphorylation of the channel proteins may be involved in electric remodeling of hypertrophic smooth muscle cells induced by partial intestinal obstruction.

Chang et al [6] reported that the RMP of smooth muscles is decreased after obstruction in mouse small intestine. In the present study, we obtained similar results. This phenomenon suggests that RMP reduction may be a common and important pathophysiological alteration in the hypertrophic smooth muscle cells induced by obstruction. It is generally accepted that voltage-dependent potassium channels are ubiquitously expressed in the whole GI tract and generate outward currents in the smooth muscle cells. This current regulates gastroineestinal motility by setting the RMP, influencing slow waves and action potential configuration [24]–[27]. As described above, the RMP became more positive in hypertrophic smooth muscle cells, so we proposed a hypothesis that K_V_ channels may be involved in the RMP alteration of hypertrophic smooth muscle cells in the obstruction model. In the present study, we found that IK_V_ density was significantly decreased in the hypertrophic smooth muscle cells. Many GI motility disorders were reported in the hypertrophic GI tract of mouse, rat and guinea pig [6]–[9]. However, the molecular mechanism of the RMP alteration is still unclear. In this regard, this finding apparently provided the first evidence to link the alteration of IK_V_ with the motility disorder in hypertrophic GI tract. In generally, IK_Vpeak_ has a more negative activation threshold (∼−50 mv) than IK_Vsustained_; that is to say IK_Vpeak_ can participate in setting RMP but not IK_Vsustained_ under resting condition [11], despite their tendency to undergo rapid inactivation at depolarized potentials. Because of this, IK_Vpeak_ may be the key player in the decrease of the RMP other than IK_Vsustained_, although the density of IK_Vsustained_ diminished. The decrease of IK_V_ can enhance the excitability of smooth muscle cell, and enhance the tension of muscle stripe and facilitate the muscle contraction. Then in the partial obstruction model, reduced IK_V_ endows hypertrophic intestinal smooth muscles with higher excitability to fit for the high tension environment.

In present study, we also observed that the value for half-activation voltage (V_0.5, act_) of IK_Vsustained_ became more positive and the value of IK_Vpeak_ showed opposite tendency in hypertrophic smooth muscle cells. These findings suggest that voltage-sensitivity of IK_Vpeak_ is increased while IK_Vsustained_ is reduced in hypertrophic smooth muscle cells. Increase of voltage sensitivity of IK_Vpeak_ can not contribute to the decrease of RMP in hypertrophic smooth muscle cells but not the activation kinetics alteration of IK_Vsustained_. To some extent. The alteration of K_V_ channel pharmacological properties also provided additional supportive evidence to comprehend the complicated IK_V_ remodeling. Under normal conditions, the direct binding of TEA or 4-AP to potassium channels blocks the ion passing the channels [28]–[30]. In our present study we found that the sensitivity of IK_Vpeak_ to 4-AP in hypertrophic cells did not change in comparison with normal and sham group but the sensitivity of IK_Vpeak_ to TEA slightly decreased (Fig. 5). Interestingly, high concentration of TEA (15 mM) blocked much more component of the IK_Vsustained_ in hypertrophic smooth muscle cells than normal and sham cells. The results suggest that the sensitivity of IK_Vsustained_ to TEA but not 4-AP significantly was up-regulated in hypertrophic smooth muscle cells induced by partial intestinal obstruction.

Base on their kinetic and pharmacological characters, it was reported that the Kv4 subfamily makes a great contribution to IK_Vpeak_ in murine myocytes of small intestine and colon [14]. And the Kv4.3 subunit is thought to be the main isoform of the Kv4 subfamily in mouse ileum [14]. Although we did not find any reports about Kv subfamily corresponding to the IK_Vsustained_ in mouse, in canine GI smooth muscle the K_V_2.2 may contribute to IK_Vsustained_ and Kv2.2 has similar kinetic and pharmacological properties with IK_Vsustained_ [15]. We examined the expressions of K_V_4.3 and K_V_2.2 to explain the molecular mechanisms of IK_V_ remodeling. Surprisingly, both K_V_4.3 and K_V_2.2 expressions were up-regulated in the hypertrophic smooth muscle tissues. It was an opposite result to decrease of IK_V_ density. In succession we further confirmed the Western blot results by using immunofluorescence experiments. Meanwhile, we stained the cell membrane to detect whether the two channel proteins were still in the plasma membrane. As we know, channel protein stability in the cell membrane is regulated by many events such as channel trafficking, protein degradation, endocytosis, and post-translational modification. Many reports indicated that over expression of K channel interacting protein (KChIP) facilitated the surface-expression Kv4 family in vitro and the KChIPs were also detected in the GI smooth muscles [14], [31], [32]. Our finding showed that the Kv4.3 and Kv2.2 proteins merged with plasma membrane and the protein quantity in the hypertrophic longitudinal muscle cell surface was much more than that in normal and sham groups. Thus, we excluded the possibility that the channel proteins stably existing in the cell surface were decreased in the hypertrophic smooth muscle cells.

Ion channels are the target of many signal pathways, including protein phosphorylation and dephosphorylation. Generally, protein kinases especially the serine/threonine kinases regulate most type of ion channels in normal or pathological conditions. Dixion et al. and Serodio et al. speculated that there were 8 possible phosphorylation sites for PKA and 14 sites for PKC in K_V_4.3 channel protein with bioinformatics method in which Thr503 was identified as an exact site for PKC in human K_V_4.3 and the phosphorylation of this site resulted in the decreasing of PKC activity in human ventricular myocytes [18], [33], [34]. Little is known about the information of Kv2.2 phosphorylation, but there are 83 serine and 52 threonine residues in Kv2.2 amino acid sequence that provide possible phosphorylation sites for protein kinases. A number of reports have suggested that Kv2.1, another Kv2 family subunit, is highly phosphorylated on numerous serine and threonine residues in mammalian neurons [35]–[37]. In the present study, we also found Kv4.3 and Kv2.2 were phosphorylated in mouse GI smooth muscles. The total phosphorylation levels of Kv4.3 and Kv2.2 were both increased in the hypertrophic smooth muscle cells compared with normal and sham groups, which implied that phosphorylation/dephosphorylation may affect the channels function in hypertrophic process. Although we can not provide direct evidence to link changes of phosphorylation level with Kv4.3 and Kv2.2 activities, in fact there is a high possibility that many protein kinases are activated during the hypertrophic process. The similar hypothesis has been proved to be true in hypertrophic cardiomyocytes of heart failing [38], [39]. Up to now, there is not enough information about regulation of Kv4.3 or Kv2.2 phosphorylation, more basic study about their upstream signaling molecules and phosphorylation sites are needed to be done especially in the GI tract. In the future work, we will try to explore the possible mechanism of Kv4.3 and Kv2.2 phosphorylation in the hypertrophic smooth muscle cells induced by partial intestinal obstruction.

In the present study, we found the current density of IK_V_ significantly decreased in the hypertrophic smooth muscle cells. Many reasons may contribute to the electric remodeling process. On one hand, IK_V_ density may be diluted by enlarging cell membrane area in hypertrophic smooth muscle cells compared with normal or sham cells. On the other hand, the electric remodeling also due to changes in K_V_ channel properties. As we know, the amplitude of a macroscopic current is governed by the product of number of channels (N), its open probability (Po) and the single channel current (I = NPoi). In this study, Western blot and immunohistochemistry results revealed that channel protein expressions were increased in hypertrophic smooth muscle tissues, so the reduction of IK_V_ density is not due to decrease of channel number. It should be noted, however, that we did not test other possible channels expression levels in the cell surface, and, hence, can not exclude the possibility that other channels protein decreased. Previous studies also reported similar phenomenon that the changes in protein expression of calcium channel did not correlate with alteration of current density in colonic inflammation model [40], [41]. As a kind of important post-translational modification, phosphorylation influences the inherent potassium channel properties including channel open probability and single channel currents. In our present study, the phosphorylation levels of serine and threonine, the K_V_4.3 and K_V_2.2 residues, were obviously up-regulated in hypertrophic smooth muscle cells which implied that the reduction of IK_V_ density may due to enhanced phosphorylation of channel proteins in hypertrophic smooth muscles. We should acknowledge that we did not do the single channel analysis to test alteration of the channel open probability and unitary amplitude of K_V_ channels in our animal model. The unitary amplitude of IK_V_ is too small to detect, only 1∼2 pA. Previous studies only detected a fast active current corresponding to IK_Vpeak_ in guinea pig colonic myocytes but not IK_Vsustained_ [11], [42].

In summary, the present study demonstrates that the two components of IK_V_ densities are significantly decreased in the intestinal obstruction-induced hypertrophic smooth muscle cells. This electric remodeling leads to more positive RMP in hypertrophic smooth muscle cells which is beneficial to maintain high excitability of hypertrophic smooth muscle to overcome the high resistance of intestinal luminal pressure induced by obstruction. Interestingly, expressions of the channel proteins corresponding to IK_V_ in the cell surface are increased in hypertrophic smooth muscle cells, which is contradictory to the decrease of IK_V_ density. Furthermore, we found that the phosphorylation levels of Kv4.3 and Kv2.2 are enhanced in the hypertrophic smooth muscles tissues, which suggest that the impaired channel function may be due to the phosphorylation of channel proteins. Future studies should explore what factors or signal pathway involved in electric remodeling, whether there is a “channelopathies” in hypertrophic gastrointestinal smooth muscle, and what is the up stream mechanism of electric remodeling mechanism of “channelopathies” in partial intestinal obstruction model.
